# Vaccine hesitancy and access to psoriasis care during the COVID‐19 pandemic: findings from a global patient‐reported cross‐sectional survey

**DOI:** 10.1111/bjd.21042

**Published:** 2022-05-03

**Authors:** Katie Bechman, Emma S. Cook, Nick Dand, Zenas Z.N. Yiu, Teresa Tsakok, Freya Meynell, Bolaji Coker, Alexandra Vincent, Herve Bachelez, Ines Barbosa, Matthew A. Brown, Francesca Capon, Claudia R. Contreras, Claudia De La Cruz, Paola Di Meglio, Paolo Gisondi, Denis Jullien, Jade Kelly, Jo Lambert, Camille Lancelot, Sinead M. Langan, Kayleigh J. Mason, Helen McAteer, Lucy Moorhead, Luigi Naldi, Sam Norton, Lluís Puig, Phyllis I. Spuls, Tiago Torres, Dominic Urmston, Amber Vesty, Richard B. Warren, Hoseah Waweru, John Weinman, Christopher E.M. Griffiths, Jonathan N. Barker, Catherine H. Smith, James B. Galloway, Satveer K. Mahil, PsoProtect study group

**Affiliations:** ^1^ Centre for Rheumatic Diseases King’s College London London UK; ^2^ Department of Medical and Molecular Genetics, School of Basic and Medical Biosciences, Faculty of Life Sciences and Medicine King’s College London London UK; ^3^ Health Data Research UK London UK; ^4^ Dermatology Centre Salford Royal NHS Foundation Trust, The University of Manchester, Manchester Academic Health Science Centre, NIHR Manchester Biomedical Research Centre Manchester UK; ^5^ St John’s Institute of Dermatology Guy’s and St Thomas’ NHS Foundation Trust and King’s College London London UK; ^6^ NIHR Biomedical Research Centre at Guy’s and St Thomas’ NHS Foundation Trust and King’s College London London UK; ^7^ Department of Dermatology AP‐HP Hôpital Saint‐Louis Paris France; ^8^ INSERM U1163, Imagine Institute for Human Genetic Diseases, Université de Paris Paris France; ^9^ Catedra de Dermatologia Hospital de Clinicas, Facultad de Ciencias Medicas, Universidad Nacional de Asuncion Paraguay; ^10^ Clinica Dermacross Santiago Chile; ^11^ St John’s Institute of Dermatology, School of Basic & Medical Biosciences, Faculty of Life Sciences & Medicine King’s College London London UK; ^12^ Section of Dermatology and Venereology University of Verona Verona Italy; ^13^ Department of Dermatology Edouard Herriot Hospital, Hospices Civils de Lyon, University of Lyon Lyon France; ^14^ Groupe de recherche sur le psoriasis (GrPso) de la Société Française de dermatologie Paris France; ^15^ Department of Dermatology Ghent University Ghent Belgium; ^16^ International Federation of Psoriasis Associations; ^17^ Faculty of Epidemiology, and Population Health London School of Hygiene and Tropical Medicine London UK; ^18^ School of Medicine Keele University Keele UK; ^19^ The Psoriasis Association Northampton UK; ^20^ Centro Studi GISED Bergamo Italy; ^21^ Psychology Department Institute of Psychiatry, Psychology and Neuroscience, King’s College London UK; ^22^ Department of Dermatology Hospital de la Santa Creu i Sant Pau, Universitat Autònoma de Barcelona Barcelona Catalonia Spain; ^23^ Department of Dermatology Amsterdam Public Health/Infection and Immunology, Amsterdam University Medical Centers Location AMC Amsterdam the Netherlands; ^24^ Department of Dermatology Centro Hospitalar do Porto Portugal; ^25^ School of Cancer and Pharmaceutical Sciences King’s College London London UK

dear editor, COVID‐19 vaccines protect against severe COVID‐19 outcomes; however, many individuals remain unvaccinated.^1^, ^2^ Vaccine hesitancy (delayed acceptance or refusal of vaccination despite service availability) threatens public health. In the UK general population, vaccine hesitancy is higher in women, younger people and ethnic minority groups.^3^, ^4^ Individuals with psoriasis, particularly those taking systemic immunosuppressants, are prioritized for COVID‐19 vaccination.^5^ However, information on vaccine hesitancy and its contributing factors in patients with psoriasis is scarce.^6^

We used data from the global patient‐reported PsoProtect*Me* survey^7^ to explore the impact of organizational and individual factors on COVID‐19 vaccine hesitancy. Data from 802 individuals with psoriasis from 89 countries were available (extracted 9 August 2021). Overall, 322 (40·1%) reported disrupted access to psoriasis care. These individuals were younger [median age 44 years, interquartile range (IQR) 33–56 vs. 54 years, IQR 42–64] and more likely to be of nonwhite ethnicity (13·8% vs. 10·2%) than those reporting no disruption. They had a shorter duration of psoriasis (median 23 years, IQR 10–36 vs. 31 years, IQR 17–44) and more severe psoriasis (6·1% vs. 2·8%). The proportion of participants taking systemic immunosuppressants was similar between those with and without disrupted access to care, but a smaller proportion with disrupted care were taking targeted immunosuppressants than those without disruption (72 of 131, 55·0% vs. 140 of 194, 72·2%).

In total, 611 (80·9%) of 755 participants had received at least one vaccine dose; 99 (16·2%) reported worsened psoriasis following vaccination, with 63 describing changes within 2 weeks. Sixty‐three (8·3%) were vaccine hesitant. These individuals were younger (median age 36, IQR 30–50 vs. 52, IQR 39–63), and more likely to be of nonwhite ethnicity (20% vs. 7·2%), live outside the UK (14% vs. 5·1%), have a numerically lower body mass index (median 24·5 kg m^−2^, IQR 21·7–28·3 vs. 26·5 kg m^−2^, 23·2–30·9) and have a shorter disease duration (median 19 years, IQR 9–32 vs. 28 years, IQR 14–42) than those who were not hesitant (Figure 1a–c). They were less likely to be taking systemic immunosuppressants (26% vs. 45·3%). The most common reasons for hesitancy were concerns regarding side‐effects, the vaccine being new and psoriasis worsening postvaccination.

**Figure 1:**
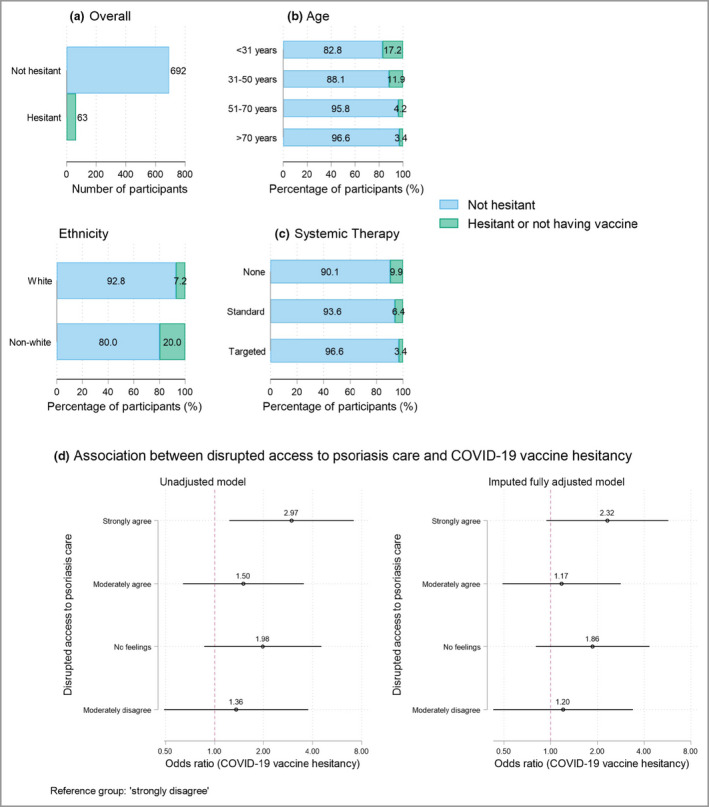
COVID‐19 vaccine hesitancy in individuals with psoriasis: (a) overall count, (b) by age and ethnicity (age < 31 years *n* = 93, 31–50 years *n* = 219, 51–70 years *n* = 261, > 70 years *n* = 58; white ethnicity *n* = 559, nonwhite ethnicity *n* = 70) and (c) by systemic immunosuppressant therapy (no therapy *n* = 406, standard therapy *n* = 110, targeted therapy *n* = 207). (d) Association between disrupted access to psoriasis care and COVID‐19 vaccine hesitancy.

In an unadjusted logistic regression model, strongly agreeing that psoriasis care was disrupted, was associated with vaccine hesitancy [compared with strongly disagreeing; odds ratio (OR) 2·97, 95% confidence interval (CI) 1·23–7·13, *P* = 0·015]. The direction of association remained after adjusting for age, sex and ethnicity, although this was not statistically significant (adjusted OR 1·90, 95% CI 0·72–5·05) (Figure 1d). In the imputed multivariate model (fitted due to missing demographic data), the association was stronger but not significant (adjusted OR 2·32, 95% CI 0·94–5·71). In total, 56 of 320 (17·5%) individuals taking standard, targeted or combination immunosuppressants were nonadherent. Nonadherence and vaccine hesitancy were not significantly associated (adjusted OR 2·96, 95% CI 0·77–11·3).

We observed an association between disrupted access to psoriasis care and COVID‐19 vaccine hesitancy, partly mediated by confounding demographic variables. Individuals feeling disenfranchized by healthcare services were more likely to be vaccine hesitant. In keeping with this finding, higher vaccine hesitancy in the general population is observed in those with negative experiences of healthcare and negative perceptions of doctors.^4^ Higher care expectations, sometimes seen in individuals with worse disease,^8^ may also be contributory. Patients taking targeted immunosuppressants were less likely to report disrupted access to care, possibly due to more frequent monitoring in secondary care, which may have been prioritized during the pandemic.

A minority (8%) of our sample reported vaccine hesitancy. This finding supports current limited data on vaccine hesitancy among patients with psoriasis.^6^ In contrast to a prior report in psoriasis,^6^ but in keeping with general population trends,^4^ hesitancy was more prominent among younger people. Our study was conducted after the COVID‐19 vaccine rollout commenced, addressing a limitation of studies characterizing intention rather than actual behaviour.^6^ In keeping with other studies, our findings also indicate greater hesitancy in individuals of nonwhite ethnicity; however, there were no differences by sex.^4^

We are unable to directly compare our global psoriasis dataset to the general population or groups with other diseases due to a lack of control samples. Participants were mostly from the UK, female and of white ethnicity, limiting generalizability. Proportionally more patients reported receiving targeted vs. standard immunosuppression, indicating ascertainment bias. Directions of associations cannot be definitively ascertained due to the cross‐sectional study design. Impacts on care and/or vaccine uptake may have been underestimated as individuals participating in health surveys may be more engaged with healthcare and vaccination services. PsoProtect*Me* was updated 1 year following its launch to include COVID‐19 vaccine hesitancy and questions regarding access to care, hence the current sample may not be representative of the original larger sample.^7^

Taken together, these data indicate that only a minority of individuals with psoriasis have vaccine hesitancy hence our findings are promising for current and future COVID‐19 vaccine uptake. Identifying individuals who are disenfranchized by healthcare services and addressing their concerns regarding COVID‐19 vaccination will help mitigate risks from the ongoing pandemic.

## Author contributions

**Katie Bechman:** Data curation (equal); formal analysis (equal); supervision (equal); visualization (equal); writing – original draft (equal). **Emma S Cook:** Formal analysis (equal); visualization (equal); writing – original draft (equal). **Nick Dand:** Conceptualization (equal); data curation (equal); investigation (equal); methodology (equal); project administration (equal); writing – review and editing (equal). **Zenas Zee Ngai Yiu:** Conceptualization (equal); investigation (equal); methodology (equal); writing – review and editing (equal). **Teresa Tsakok:** Data curation (equal); investigation (equal); methodology (equal); project administration (equal); resources (equal); writing – review and editing (equal). **Freya Meynell:** Project administration (equal). **Bolaji Coker:** Data curation (equal); project administration (equal); resources (equal); software (equal). **Alexandra Vincent:** Data curation (equal); project administration (equal). **Herve Bachelez:** Investigation (equal); methodology (equal); writing – review and editing (equal). **Ines Barbosa:** Project administration (equal). **Matthew Brown:** Conceptualization (equal); funding acquisition (equal); investigation (equal); methodology (equal); resources (equal); supervision (equal); writing – review and editing (equal). **Francesca Capon:** Investigation (equal); methodology (equal); writing – review and editing (equal). **Claudia Contreras:** Investigation (equal); methodology (equal); writing – review and editing (equal). **Claudia De la Cruz:** Investigation (equal); writing – review and editing (equal). **Paola Di Meglio:** Investigation (equal); methodology (equal); writing – review and editing (equal). **Paolo Gisondi:** Investigation (equal); methodology (equal); writing – review and editing (equal). **Denis Jullien:** Investigation (equal); methodology (equal); writing – review and editing (equal). **Jade Kelly:** Project administration (equal). **Jo Lambert:** Investigation (equal); methodology (equal); writing – review and editing (equal). **Camille Lancelot:** Project administration (equal). **Sinead Langan:** Conceptualization (equal); investigation (equal); methodology (equal); writing – review and editing (equal). **Kayleigh J Mason:** Conceptualization (equal); data curation (equal); investigation (equal); methodology (equal); writing – review and editing (equal). **Helen McAteer:** Conceptualization (equal); data curation (equal); investigation (equal); resources (equal); writing – review and editing (equal). **Lucy Moorhead:** Project administration (equal). **Luigi Naldi:** Investigation (equal); methodology (equal); writing – review and editing (equal). **Sam Norton:** Investigation (equal); methodology (equal); writing – review and editing (equal). **Lluís Puig:** Investigation (equal); methodology (equal); writing – review and editing (equal). **Phyllis I. Spuls:** Investigation (equal); methodology (equal); writing – review and editing (equal). **Tiago Torres:** Investigation (equal); methodology (equal); writing – review and editing (equal). **Dominic Urmston:** Project administration (equal); resources (equal). **Amber Vesty:** Project administration (equal). **Richard B Warren:** Investigation (equal); methodology (equal); writing – review and editing (equal). **Hoseah Waweru:** Investigation (equal); methodology (equal). **John Weinman:** Investigation (equal); methodology (equal); writing – review and editing (equal). **Christopher Ernest, Maitland Griffiths:** Conceptualization (equal); investigation (equal); methodology (equal); resources (equal); supervision (equal); writing – review and editing (equal). **Jonathan N W N Barker:** Conceptualization (equal); investigation (equal); methodology (equal); resources (equal); supervision (equal); writing – review and editing (equal). **Catherine H. Smith:** Conceptualization (equal); data curation (equal); funding acquisition (equal); investigation (equal); methodology (equal); project administration (equal); resources (equal); software (equal); supervision (equal); writing – review and editing (equal). **James Galloway:** Conceptualization (equal); formal analysis (equal); investigation (equal); methodology (equal); resources (equal); supervision (equal); writing – review and editing (equal). **Satveer Mahil:** Conceptualization (equal); data curation (equal); investigation (equal); methodology (equal); project administration (equal); resources (equal); writing – original draft (equal); writing – review and editing (equal).

## Supporting information


**Appendix S1** Full list of affiliations.

**Appendix S2** Funding sources.

**Appendix S3** Conflicts of interest.
