# Supplementary material for: Vaccine hesitancy and access to psoriasis care during the COVID‐19 pandemic: findings from a global patient‐reported cross‐sectional survey
Source: Br J Dermatol. 2022 May 3;187(2):254–6. doi: 10.1111/bjd.21042 (PMC9545500; doi:10.1111/bjd.21042)
Supplement: Supplementary file 1 — Appendix S1 Full list of affiliations. [file BJD-187-254-s002.docx]

Katie Bechman,^1^ Emma S. Cook,^1^ Nick Dand,^2,3^ Zenas Z.N. Yiu,^4^ Teresa Tsakok,^5^ Freya Meynell,^5^ Bolaji Coker,^6^ Alexandra Vincent,^6^ Herve Bachelez,^7,8^ Ines Barbosa,^5^ Matthew A. Brown,^1,6^ Francesca Capon,^2,6^ Claudia R. Contreras,^9^ Claudia De La Cruz,^10^ Paola Di Meglio,^6,11^ Paolo Gisondi,^12^ Denis Jullien,^13,14^ Jade Kelly,^4^ Jo Lambert,^15^ Camille Lancelot,^16^ Sinead M. Langan,^5,17^ K.J. Mason,^4,18^ Helen McAteer,^19^ Lucy Moorhead,^5^ Luigi Naldi,^20^ Sam Norton,^21^ Lluís Puig,^22^ Phyllis I. Spuls,^23^ Tiago Torres,^24^ Dominic Urmston,^19^ Amber Vesty,^19^ Richard B. Warren,^4^ Hoseah Waweru,^16^ John Weinman,^25^ Christopher E.M. Griffiths,^4^ Jonathan N. Barker,^6,11^ Catherine H. Smith,^5,6^ James B. Galloway^1^ and Satveer K. Mahil;^5,6^ on behalf of the PsoProtect study group

^1^Centre for Rheumatic Diseases, King’s College London, London, UK; ^2^Department of Medical and Molecular Genetics, School of Basic and Medical Biosciences, Faculty of Life Sciences and Medicine, King’s College London, London, UK; ^3^Health Data Research UK, London, UK; ^4^Dermatology Centre, Salford Royal NHS Foundation Trust, The University of Manchester, Manchester Academic Health Science Centre, NIHR Manchester Biomedical Research Centre, Manchester, UK; ^5^St John’s Institute of Dermatology, Guy’s and St Thomas’ NHS Foundation Trust and King’s College London, London, UK; ^6^NIHR Biomedical Research Centre at Guy’s and St Thomas’ NHS Foundation Trust and King’s College London, London, UK; ^7^Department of Dermatology, AP-HP Hôpital Saint-Louis, Paris, France; ^8^INSERM U1163, Imagine Institute for Human Genetic Diseases, Université de Paris, Paris, France; ^9^Catedra de Dermatologia, Hospital de Clinicas, Facultad de Ciencias Medicas, Universidad Nacional de Asuncion, Paraguay; ^10^Clinica Dermacross, Santiago, Chile; ^11^St John’s Institute of Dermatology, School of Basic & Medical Biosciences, Faculty of Life Sciences & Medicine, King’s College London, London, UK; ^12^Section of Dermatology and Venereology, University of Verona, Verona, Italy; ^13^Department of Dermatology, Edouard Herriot Hospital, Hospices Civils de Lyon, University of Lyon, Lyon, France; ^14^Groupe de recherche sur le psoriasis (GrPso) de la Société Française de dermatologie, Paris, France; ^15^Department of Dermatology, Ghent University, Ghent, Belgium; ^16^International Federation of Psoriasis Associations; ^17^Faculty of Epidemiology, and Population Health, London School of Hygiene and Tropical Medicine, London, UK; ^18^School of Medicine, Keele University, Keele, UK; ^19^The Psoriasis Association, Northampton, UK; ^20^Centro Studi GISED, Bergamo, Italy; ^21^Psychology Department, Institute of Psychiatry, Psychology and Neuroscience, King’s College London, UK; ^22^Department of Dermatology, Hospital de la Santa Creu i Sant Pau, Universitat Autònoma de Barcelona, Barcelona, Catalonia, Spain; ^23^Department of Dermatology, Amsterdam Public Health/Infection and Immunology, Amsterdam University Medical Centers, Location AMC, Amsterdam, the Netherlands; ^24^Department of Dermatology, Centro Hospitalar do Porto, Portugal; ^25^School of Cancer and Pharmaceutical Sciences, King’s College London, London, UK
