# Supplementary material for: Vaccine hesitancy and access to psoriasis care during the COVID‐19 pandemic: findings from a global patient‐reported cross‐sectional survey
Source: Br J Dermatol. 2022 May 3;187(2):254–6. doi: 10.1111/bjd.21042 (PMC9545500; doi:10.1111/bjd.21042)
Supplement: Supplementary file 2 — Appendix S2 Funding sources. [file BJD-187-254-s003.docx]

Funding sources

We acknowledge financial support from the Department of Health via the National Institute for Health Research (NIHR) Biomedical Research Centre based at Guy’s and St Thomas’ NHS Foundation Trust and King’s College London, the NIHR Manchester Biomedical Research Centre and the Psoriasis Association. The views expressed are those of the author(s) and not necessarily those of the NHS, the NIHR, or the Department of Health and Social Care. SKM is funded by a Medical Research Council (MRC) Clinical Academic Research Partnership award (MR/T02383X/1). ND is funded by Health Data Research UK (MR/S003126/1), which is funded by the UK MRC, Engineering and Physical Sciences Research Council; Economic and Social Research Council; Department of Health & Social Care (England); Chief Scientist Office of the Scottish Government Health and Social Care Directorates; Health and Social Care Research and Development Division (Welsh Government); Public Health Agency (Northern Ireland); British Heart Foundation; and Wellcome Trust. ZZNY is funded by a NIHR Academic Clinical Lectureship through the University of Manchester. CEMG is a NIHR Emeritus Senior Investigator and is funded in part by the MRC (MR/101 1808/1). CEMG and RBW are in part supported by the NIHR Manchester Biomedical Research Centre. SML is supported by a Wellcome senior research fellowship in clinical science (205039/Z/16/Z); this research was funded in whole or in part by the Wellcome Trust [205039/Z/16/Z]. For the purpose of Open Access, the author has applied a CC BY public copyright licence to any Author Accepted Manuscript (AAM) version arising from this submission. SML is also supported by Health Data Research UK (grant no. LOND1), which is funded by the UK MRC, Engineering and Physical Sciences Research Council, Economic and Social Research Council, Department of Health and Social Care (England), Chief Scientist Office of the Scottish Government Health and Social Care Directorates, Health and Social Care Research and Development Division (Welsh Government), Public Health Agency (Northern Ireland), British Heart Foundation and Wellcome Trust.
