# Supplementary material for: Vaccine hesitancy and access to psoriasis care during the COVID‐19 pandemic: findings from a global patient‐reported cross‐sectional survey
Source: Br J Dermatol. 2022 May 3;187(2):254–6. doi: 10.1111/bjd.21042 (PMC9545500; doi:10.1111/bjd.21042)
Supplement: Supplementary file 3 — Appendix S3 Conflicts of interest. [file BJD-187-254-s001.docx]

Conflicts of interest

Nothing to disclose: Dr Bechman, Ms Cook, Dr Dand, Prof. Langan, Dr Norton, Dr Tsakok, Dr Yiu, Dr De La Cruz, Dr Contreras, Ms. Vesty, Ms. Vincent, Mr Bola Coker, Ms. Meynell, Ms Kelly, Ms Lancelot, Dr Lambert, Prof. Brown, Prof. Naldi.

Prof. Barker reports grants and personal fees from AbbVie, grants and personal fees from Novartis, grants and personal fees from Lilly, grants and personal fees from J&J, from null, during the conduct of the study.

Prof. Griffiths reports grants and personal fees from AbbVie, grants from Amgen, grants from BMS, grants and personal fees from Janssen, grants from LEO, grants and personal fees from Novartis, grants from Pfizer, grants from Almirall, grants and personal fees from Lilly, grants and personal fees from UCB Pharma, outside the submitted work.

Prof. Jullien reports personal fees and non-financial support from AbbVie, personal fees and non-financial support from Novartis, personal fees and non-financial support from Janssen-Cilag, personal fees and non-financial support from Lilly, personal fees and non-financial support from Leo-Pharma, personal fees and non-financial support from MEDAC, personal fees and non-financial support from Celgene, personal fees from Amgen, outside the submitted work.

Dr Capon reports consultancy fees from AnaptysBio, grants from Boheringer-Ingelheim, outside the submitted work.

Prof. Bachelez reports personal fees from AbbVie, personal fees from Janssen, personal fees from LEO Pharma, personal fees from Novartis, personal fees from UCB, personal fees from Almirall, personal fees from Biocad, personal fees from Boehringer Ingelheim, personal fees from Kyowa Kirin, personal fees from Pfizer, outside the submitted work.

Prof. Gisondi reports personal fees from AbbVie, Amgen, Eli Lilly, Janssen, Novartis, Pierre Fabre, Sandoz, UCB, outside the submitted work.

Dr Galloway reports personal fees from AbbVie, personal fees from Sanofi, personal fees from Novartis, personal fees from Pfizer, grants from Eli Lilly, personal fees from Janssen, personal fees from UCB, outside the submitted work.

Prof. Weinmann has presented talks for AbbVie, Abbott, Bayer, Chiesi, Boehringer Ingelheim, Roche and Merck.

Dr Mason reports personal fees from LEO Pharma and Novartis, outside the submitted work.

Ms. Moorhead reports personal fees from AbbVie, personal fees from Celgene, personal fees from Janssen, personal fees from LEO Pharma, personal fees from Novartis, personal fees from UCB, outside the submitted work.

Dr Puig reports grants and personal fees from AbbVie, grants and personal fees from Almirall, grants and personal fees from Amgen, grants and personal fees from Boehringer Ingelheim, personal fees from Bristol Myers Squibb, personal fees from Fresenius-Kabi, grants and personal fees from Janssen, grants and personal fees from Lilly, personal fees from Mylan, grants and personal fees from Novartis, personal fees from Pfizer, personal fees from Sandoz, personal fees from Sanofi, personal fees from Samsung-Bioepis, grants and personal fees from UCB, outside the submitted work.

Dr Mahil reports departmental income from AbbVie, Almirall, Eli Lilly, Janssen-Cilag, Novartis, Sanofi, UCB, outside the submitted work.

Dr Di Meglio reports grants and personal fees from UCB, personal fees from Novartis, personal fees from Janssen, outside the submitted work.

Prof. Warren reports grants and personal fees from AbbVie, grants and personal fees from Celgene, grants and personal fees from Eli Lilly, grants and personal fees from Novartis, personal fees from Sanofi, grants and personal fees from UCB|, grants and personal fees from Almirall, grants and personal fees from Amgen, grants and personal fees from Janssen, grants and personal fees from Leo, grants and personal fees from Pfizer, personal fees from Arena, personal fees from Avillion, personal fees from Bristol Myers Squibb, personal fees from Boehringer Ingelheim, outside the submitted work.

Prof. Smith reports grants from AbbVie, Sanofi, Novartis, and Pfizer and through consortia with multiple academic partners (psort.org.uk, BIOMAP-IMI.eu), outside the submitted work.

Dr Torres reports grants and personal fees from AbbVie, Almirall, Amgen, Arena Pharmaceuticals, Biogen, Biocad, Boehringer Ingelheim, Bristol Myers Squibb, Celgene, Eli Lilly, Janssen, LEO Pharma, MSD, Novartis, Pfizer, Samsung-Bioepis, Sandoz, during the conduct of the study.

Dr Waweru is on the Board of the International Federation of Psoriasis Associations who have received grants from AbbVie, Almirall, Amgen, Bristol Meyers Squibb, Boehringer Ingelheim, Celgene, Janssen, LEO Pharma, Eli Lilly, Novartis, Sun Pharma, Pfizer, and UCB, outside the submitted work.

Mr Urmston reports grants from Almirall, grants from AbbVie, grants from Amgen, grants from Celgene, grants from Dermal Laboratories, grants from Eli Lilly, grants from Janssen, grants from LEO Pharma, grants from T and R Derma, grants from UCB, outside the submitted work.

Ms. McAteer reports grants from AbbVie, grants from Almirall, grants from Amgen, grants from Celgene, grants from Dermal Laboratories, grants from Eli Lilly, grants from Janssen, grants from LEO Pharma, grants from UCB, grants from T and R Derma, outside the submitted work.

Prof. Spuls has done consultancies in the past for Sanofi 111017 and AbbVie 041217 (unpaid), received a departmental independent research grant for TREAT NL registry LEO Pharma December 2019; is involved in performing clinical trials with many pharmaceutical industries that manufacture drugs used for the treatment of diseases such as psoriasis and atopic dermatitis, for which financial compensation is paid to the department/hospital; and is chief investigator of the systemic and phototherapy atopic eczema registry (TREAT NL) for adults and children, as well as one of the main investigators of the SECURE-AD registry.
